# The effect of hydraulic flowback and produced water on gill morphology, oxidative stress and antioxidant response in rainbow trout (*Oncorhynchus mykiss*)

**DOI:** 10.1038/srep46582

**Published:** 2017-04-20

**Authors:** Tamzin A. Blewett, Alyssa M. Weinrauch, Perrine L. M. Delompré, Greg G. Goss

**Affiliations:** 1 Department of Biological Sciences, University of Alberta, 11455 Saskatchewan Drive, Edmonton, Alberta, T6G 2E9, Canada; 2 National Institute for Nanotechnology, 11421 Saskatchewan Drive, Edmonton, Alberta, T6G 2M9, Canada

## Abstract

Hydraulic fracturing fluid are complex mixtures containing high concentrations of salts (up to 330,000 ppm), organic, and metal contaminants. However, little data exist on the potential mechanisms of toxicity of these flowback and produced wastewaters (FPW) on aquatic biota. Juvenile rainbow trout were exposed to either control, FPW (2.5 or 7.5%), FPW that had been treated with activated charcoal (AC), or a custom salt-matched control (SW; replicating only the salt content of FPW) for 48 hours. Gill histology revealed decreases in interlamellar cell mass (ILCM) and mean lamellar length in all treatments (FPW, AC and SW) compared to control, indicative of hyperosmotic stress. Liver CYP1A1 activity was significantly elevated by 7.5-fold in the FPW 7.5% treatment only, indicative of Phase I metabolism. Superoxide dismutase activity significantly decreased in the gills to all treatments with the lowest activity occurring in the 7.5% FPW group. Catalase activity increased in liver with the highest values noted in fish exposed to 7.5% FPW. No changes were observed with respect to glutathione-S-transferase, while increased lipid peroxidation was only observed in both FPW treatments (2.5, 7.5%). These data suggest a characteristic signature of FPW impact which may help in risk assessment and biomonitoring of FPW spills.

The process of oil and gas recovery from geological formations of low permeability is termed hydraulic fracturing^1^. In 2012, hydraulic fracturing extraction accounted for 385 billion m^3^/yr of natural gas. Hydraulic fracturing involves pumping pressurized water (upwards of 69,000 kPa) into formations rich in oil and gas^1^. Over time, the hydraulic fracturing fluid returns to the surface as pressure is released, bringing with it components associated with the geology of the formation and the entrapped fluids in the pore spaces. This fluid is called flowback and produced water (FPW). The difference between flowback and produced water is time spent in the well; flowback usually refers to return of injected fluids, while produced water is formation water that is high in gas and oil^1^. FPW is chemically complex, temporally heterogeneous and can contain biocides, polycyclic aromatic hydrocarbons (PAHs), radioisotopes, salts (sodium, calcium, magnesium and potassium) and metals^1^,^2^,^3^. Given its complexity and concentration of potentially toxic components, spills of FPW have the capacity for significant impacts on the environment.

There are stakeholder concerns regarding water use in hydraulic fracturing and its management^4^ as well as technical and environmental challenges regarding the disposal of FPW^5^,^6^,^7^,^8^,^9^,^10^. In the province of Alberta, Canada, an estimated 2500 FPW spills occurred from 2005 to 2012, with more than 113 of those spills entering directly into freshwater lakes and streams^11^. Thus, an understanding of the toxicological properties and effects of FPW is critical for establishing best management practices, proper risk assessment, spill response and remediation of FPW spill sites.

One mechanism by which FPW may cause toxic effects in biota exposed to spills is via oxidative stress. Salts, metals and organics are core components of most FPWs, and are all known to induce oxidative stress in fish^12^. Oxidative stress arises due to an imbalance between the production of reactive oxygen species (ROS) and antioxidant defence mechanisms. The generation of ROS is a natural consequence of molecular oxygen being reduced to water in the electron transport chain^12^. At high concentrations ROS can cause chain reactions leading to damage to major biomolecules, including^13^: (*i*) reactions with nucleic acids, nucleotides, polysaccharides, (*ii*) covalent binding to membrane components (i.e. lipid, proteins), (*iii*) oxidation of membrane proteins and lipids. For the most part, ROS are scavenged by antioxidant defence mechanisms, including enzymes such as superoxide dismutase, catalase, and glutathione peroxidases. However, any factor that disturbs the balance between production and clearing of ROS in biological systems may cause excess oxidative stress^12^. To date, there is no study that has specifically examined antioxidant enzyme impacts arising from FPW exposure.

An additional sensitive end-point of toxicant exposure for fish is change in the morphology of the gill epithelium. The gill is the primary tissue involved in respiration, osmoregulation, acid/base regulation and nitrogen excretion in fish^14^, and comprises a large surface area that is in direct contact with a contaminated medium. Previous studies have shown changes in fish gill morphology in response to waterborne metals^15^, organic toxicants^16^ and elevated salts^17^,^18^. There is evidence that hydrocarbon-rich wastewaters similar to those of FPW (i.e. those associated with oil-sands production) may also cause changes in fish gill morphology^19^.

The current study is the first to investigate the effects of FPW on tissue oxidative stress (liver and gill) and gill histology following an acute exposure (48 h) to FPW in juvenile rainbow trout (*Oncorhynchus mykiss*). Rainbow trout are a standard toxicological model organism and often used in both regulatory testing and risk assessment. They are highly sensitive to aquatic contaminants^20^, and an economically and culturally important fish species, inhabiting North American waters at risk of FPW spills^21^.

## Methods

### Animal Care

Rainbow trout fingerlings were obtained as fertilized eggs from Raven Brood Trout Station (Caroline, Alberta, Canada), and held for 1 year at the Department of Biological Sciences at the University of Alberta until they had reached a size of 7.3 g ± 1.1. Fish were housed in a 450-L flow-through system using dechlorinated city of Edmonton tap water (moderately hard: [Na^+^] = 14.6 ppm, [Ca^2+^] = 55.9 ppm, [Mg^2+^] = 15.3 ppm, [K^+^] = 2.5 ppm, titratable alkalinity ≈ 119 mg/L as CaCO_3_, pH ≈ 7.6, hardness ≈ 180 mg/L as CaCO_3_, conductivity ≈ 385 μS/cm). Fish were maintained in waters with a temperature of 13 ± 1 °C under a light:dark cycle of 14:10. This research is in accordance with all relevant guidelines and regulations. All animal use and experiments were approved by the University of Alberta and the Canadian Council on Animal Care Committee under protocol AUP00001334.

### Water Chemistry

The FPW sample tested was a 10 day sample from hydraulic fracturing of the Devonian-aged Duvernay Formation (Fox Creek, Alberta, Canada) provided by Encana Corporation. In this study, FPW refers to the original, raw sample containing all sediment and suspended particles. Activated-charcoal-treated (AC) and saltwater–matched controls (SW) were also prepared as described in previous studies^3^,^4^. The AC treatment therefore contains all the components of the original FPW except those bound by activated charcoal (i.e. organic components and some metals). The SW treatment was made up to resemble the salt load of FPW by addition of lab salts to nanopure water (salts used were NaCl, MgCl_2_, KCl, CaCl_2_; Sigma Aldrich). For example, the 2.5% FPW sample (and thus the 2.5% SW sample) had a salinity of ~4.5 ppt^2^). All samples were stored in the dark at room temperature prior to use. The water chemistry of the exposure samples has been characterized in companion manuscripts^2^,^3^,^4^.

### Exposures

Fish were housed in 8-L tanks with constant aeration, and were fasted 24 hours prior to experimentation. The tanks were situated in a flow-through recirculating water bath to keep temperature constant at 13 °C. At the start of the experimental procedure fish were placed in either a control tank or tanks dosed with FPW to give final dilutions of either 2.5% FPW, 7.5% FPW, activated charcoal (7.5%) or saltwater-matched control (7.5%). After 24 h, 80% of the water was replaced with water of identical composition, and at 48 h, fish were euthanized by spinal transection, before gills and liver were removed for enzyme and histological analysis.

### Gill Histology

Gills were excised from three randomly selected rainbow trout in each treatment group and fixed in a 4% paraformaldehyde, 2.5% glutaraldehyde, 0.05 mmol L^−1^ sodium cacodylate buffer (pH 7.4) on ice (4 **°**C) for 3 h. Samples underwent an ethanol dehydration series prior to paraffin wax embedding. Tissues were trimmed and sectioned (7 μm) and post-stained with haematoxylin and eosin (H&E) prior to visualization on a Zeiss Scope A1 with image capture using an optronic camera. Representative digital images containing 10+ adjacent lamella were randomly selected and ImageJ software (National Institute of Health) was used to calculate the lamellar width, lamellar length, interlamellar cell mass (ILCM) and ILCM:lamellar length (as described previously by^18^,^22^.

### Oxidative Stress Parameters

Gill and liver tissue were snap-frozen in liquid nitrogen, then ground to a fine powder under liquid nitrogen using a mortar and pestle, and placed in −80 °C until assayed. All assays for antioxidant enzymes (superoxide dismutase, catalase and glutathione-S-transferase) were performed on both gill and liver using commercially-available kits (Cayman Chemical, Burlington, ON). For the assays described below, all measurements were made on a 96-well plastic plate read on a Versamax Molecular Devices microplate reader using Software Max Pro 5. All assays were normalized to protein content with protein determined *via* a bicinchoninic acid (BCA) Protein Assay Kit using manufacturer’s protocol (Thermo Scientific, USA).

For superoxide dismutase (SOD) activity determination, ground tissues were placed in buffer containing 20 mM HEPES, 1 mM EGTA, 210 mM mannitol and 70 mM sucrose (Sigma Aldrich, Oakville, ON), corrected to a pH of 7.2 and homogenized using a Potter-Elvehjem homogenizer. The homogenate was then centrifuged at 1,500 g for 5 min at 4 °C and supernatant was used for SOD determination, using the reaction of tetrazolium salt for detection of superoxide radicals, at a wavelength of 450 nm. One unit (U) of SOD is defined as the amount of enzyme needed to exhibit 50% dismutation of the superoxide radical.

Catalase activity was measured *via* the decomposition of hydrogen peroxide over time, measured at a wavelength of 540 nm. Gill and liver tissue were homogenized in a buffer containing 50 mM potassium phosphate, 1 mM EDTA (Sigma Aldrich, Oakville, ON) at a pH 7.0. After homogenization, tissues were centrifuged for 10,000 g for 15 min at 4 °C, and the supernatant was assayed for catalase activity.

Glutathione-S-transferase (GST) activity was measured *via* the conjugation of 1, chloro-2,4 dinitrobenzene (CDNB) with reduced glutathione, measured at a wavelength of 340 nm. Tissues were homogenized in a buffer containing 100 mM potassium phosphate, and 2 mM EDTA (Sigma Aldrich, Oakville ON) at a pH of 7.0. Samples were then centrifuged at 10,000 g for 15 min at 4 °C, and the assay conducted on the resulting supernatant.

A Thiobarbituric Acid Reactive Substances (TBARS) assay was used to quantify lipid peroxidation^23^. Briefly, tissue samples were homogenized in phosphate buffer and centrifuged at 1,000 g for 1 min. Once centrifuged, 130 μL of homogenate supernatant was removed and diluted with 455 μL of phosphate buffer. To each sample, 32.5 μL of butylated hydroxytoluene (1 mM) was added to prevent further oxidation of samples, followed by 162.5 μL of trichloroacetic acid (TCA) (50%) to precipitate out interfering proteins. Samples were centrifuged at 13,000 g for 2 min and 120 μL of supernatant was transferred to a 96-well microplate, followed by 75 μL of thiobarbituric acid (1.3%, dissolved in 0.3% NaOH). A standard curve was generated using 1,1,3,3-tetraethoxypropane (0–25 μM) on the same plate (Sigma, USA). The microplate was incubated at 80 °C for 60 min and fluorescence was recorded at 531 and 572 nm (excitation and emission, respectively).

### Hepatic EROD Assay

Hepatic 7-ethoxy-resorufin–O-deethylase (EROD) activity, a marker of cytochrome P450 activity, was determined *via* a modified method^24^ Liver tissue was homogenized in ice-cold KCl-HEPES buffer (0.15 M KCl, 0.02 M HEPES, pH 7.5) using a Potter-Elvehjem homogenizer. The homogenate was centrifuged at 10,000 g (4 °C) for 20 min, and the S-9 supernatant fraction was collected and used directly in the EROD assay. Aliquots of cold S-9 hepatic fractions (40 μL) were added to a 96-well plate, followed by 200 μL of cold EROD buffer (0.1 M Tris-HCl, 1 mM EDTA, 2.5 μM 7-ethoxyresorufin, pH 7.4). The reaction was initiated by the addition of 10 μL of fresh NADPH solution (5 mM). Incubation was performed at room temperature in the dark with gentle shaking for 20 min. Following incubation, fluorescence was measured at 535 and 590 nm (excitation and emission respectively) using a Victor3V 1420 Multilabel Counter (Perkin Elmer, MA). EROD activity was normalized to S-9 fraction protein content using a BCA protein assay kit (Thermo Scientific, USA) and signal activity read as picomole of resorufin per mg protein per minute, from a resorufin standard curve (0–1000 pM). Dilutions of sample homogenate supernatants were made accordingly to yield approximately equal protein values (±1000 μg/mL) amongst samples after the phosphate buffer dilution step. Values are expressed as relative fold change from control.

### Statistics

A one-way ANOVA followed by a Tukey post hoc test was performed for all data using either SigmaPlot version 11.0 with SigmaStat version 3.5 integration (Systat Software Ind. San Jose CA, USA) or GraphPad Prism 6.0 (Graphpad Software, San Diego, USA). All data have been expressed as means ± SEM (standard error of the mean of all replicates). Significance for all tests was accepted at α = 0.05.

## Results

### Water Chemistry

The water chemistry for the exposures has been characterized in previous manuscripts^2^,^3^,^4^. The data therein shows that the FPW was highly elevated in salts (sodium 59, 500 mg/L; chloride 107, 000 mg/L), metals (zinc 1.24 mg/L), and organics (fluorene; 294 ng/L; 3, 6 dimethylphenanthrene, 224.5 ng/L).

### Gill Histology

**Figure 1:**
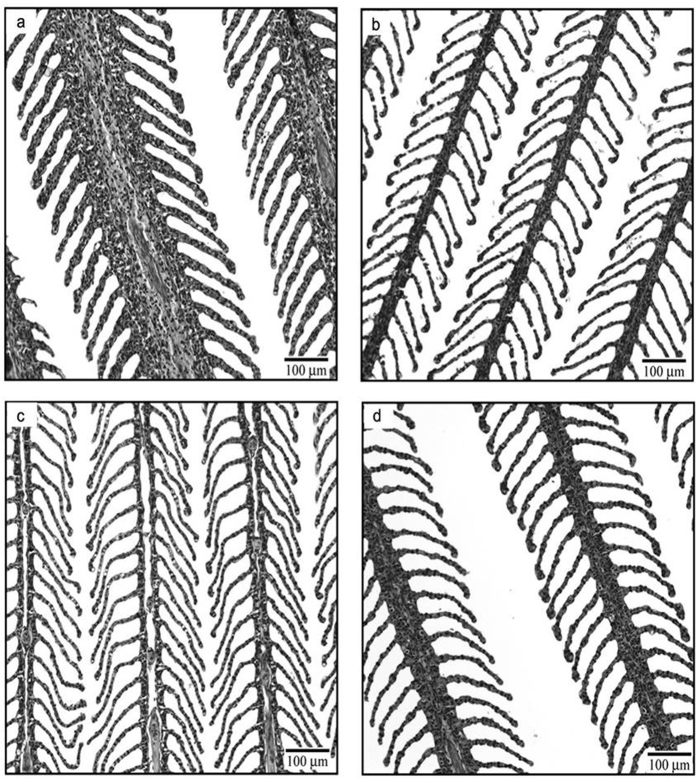
Hematoxylin and Eosin stained light micrographs of representative gills from rainbow trout exposed to control water (**A**), activated charcoal treated water (**B**), seawater-matched controls (**C**) and a 7.5% dilution of flow-back and produced water (**D**).

**Figure 2:**
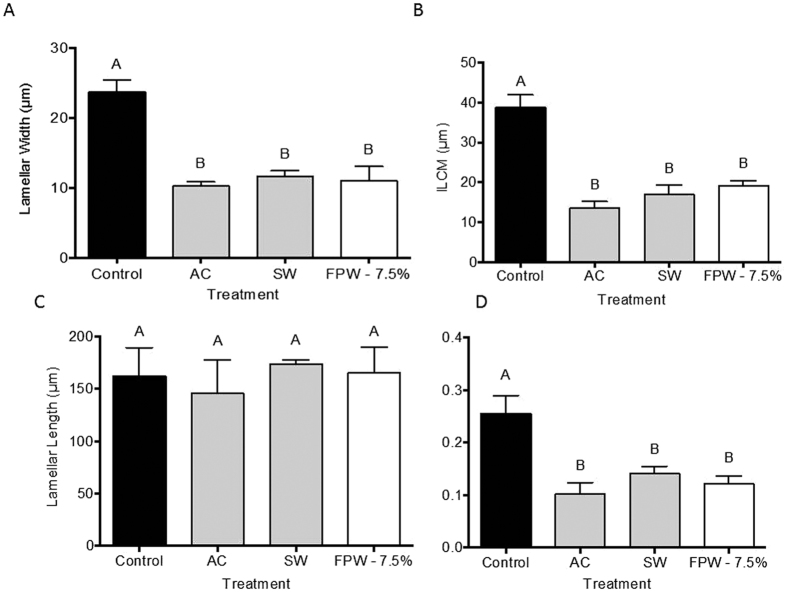
Effect of an acute exposure to control, activated charcoal (AC), saltwater-matched (SW) or 7.5% flowback and produced water (FPW) on rainbow trout gill: (**A**) lamellar width, (**B**) interlamellar cell mass (ILCM), (**C**) lamellar length, and (**D**) ratio of interlamellar mass to lamellar length. Values are mean + S.E.M (N = 3). Letters that are different denote significant differences from control (α = 0.05).

Histology of the gills revealed marked differences between control (unexposed) rainbow trout and those exposed to any of the treatment groups (AC, SW, 7.5% FPW). Mean lamellar widths from each treatment (AC, SW, 7.5% FPW) were significantly reduced (p < 0.05, N = 3) in comparison to control lamellar width (Figs 1A–D and 2A). Similarly, measurements of ILCM in gills from fish exposed to AC, SW, and FPW were significantly lower (p < 0.05, N = 3) than in control rainbow trout gills (38.6 μm; Figs 1A–D and 2B). However, no significant differences were observed between any of the treatment groups for lamellar length, which ranged from 145.5 to 173.7 μm (Figs 1A–D and 2C).

### Hepatic EROD

**Figure 3:**
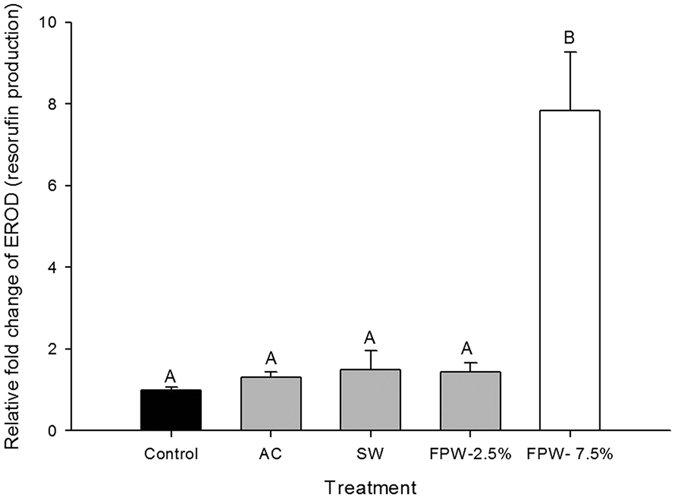
EROD activity in the liver of rainbow trout after a 48 h acute exposure to control, activated charcoal treated water (AC), a salt-matched control (SW), or flowback and produced water (FPW- 2.5% and 7.5%). Bars represent means ( ± S.E.M) of 6 to 9 replicates. Bars sharing letters are not significantly (α = 0.05).

Hepatic EROD induction was observed in the 7.5% FPW concentration only, with an almost 8-fold induction of resorufin production compared to control tissues (p < 0.05, N = 6–9; Fig. 3). There was no induction of resorufin production in either the AC, SW or 2.5% FPW treatment groups.

### Oxidative Stress Enzymes

**Figure 4:**
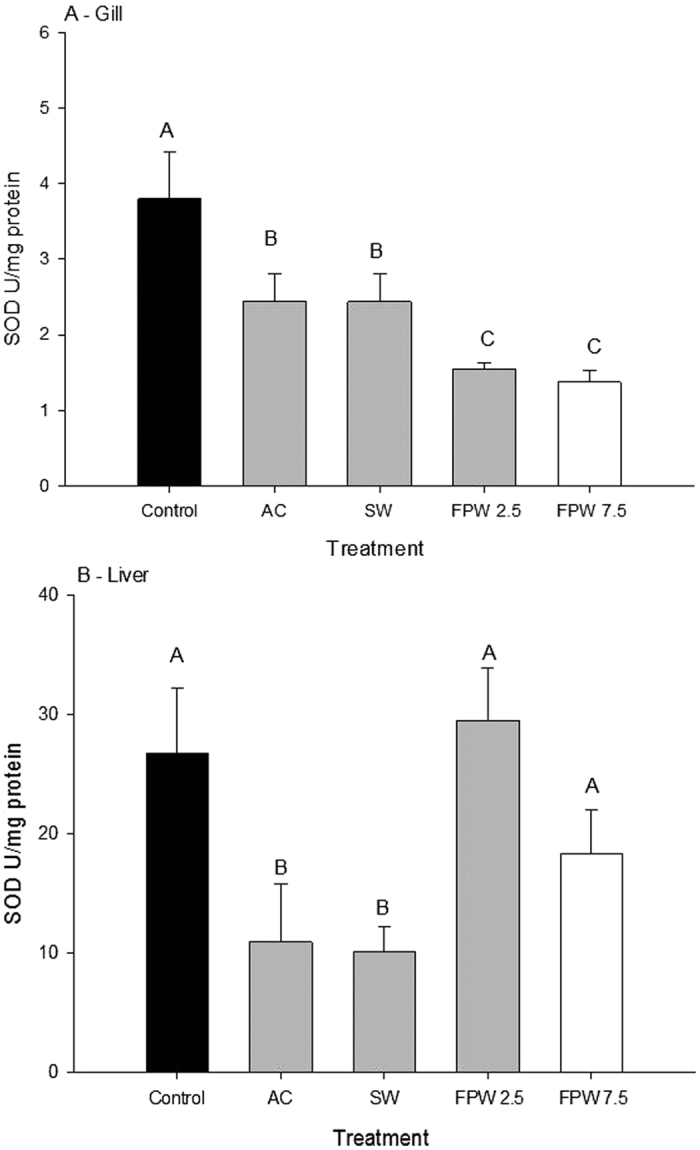
Superoxide dismutase (SOD) activity in the gill (**A**) or liver (**B**) of rainbow trout after an acute 48 h exposure to control, activated charcoal treated water (AC), a salt matched control (SW), and flow-back and produced water (FPW 2.5% and 7.5%). Bars represent means (±S.E.M) of 6 to 9 replicates. Bars sharing letters are not significantly (α = 0.05).

Branchial SOD activity was decreased significantly in all treatments relative to control gill tissue. For example, the 2.5% and 7.5% FPW treatments decreased SOD activity from 3.8 U/mg protein in the control to 1.5 and 1.3 U/mg protein, respectively. These treatments were also significantly decreased in comparison to the AC and SW treatments (p < 0.05, N = 6–9; Fig. 4A), which were in turn significantly lower than control SOD activity. However, liver tissue did not follow the same pattern of SOD activity as the gills. Exposure to AC and SW treatments significantly decreased liver SOD activity by 50%, while no significant changes were observed following exposure to either 2.5% or 7.5% FPW (Fig. 4B).

**Figure 5:**
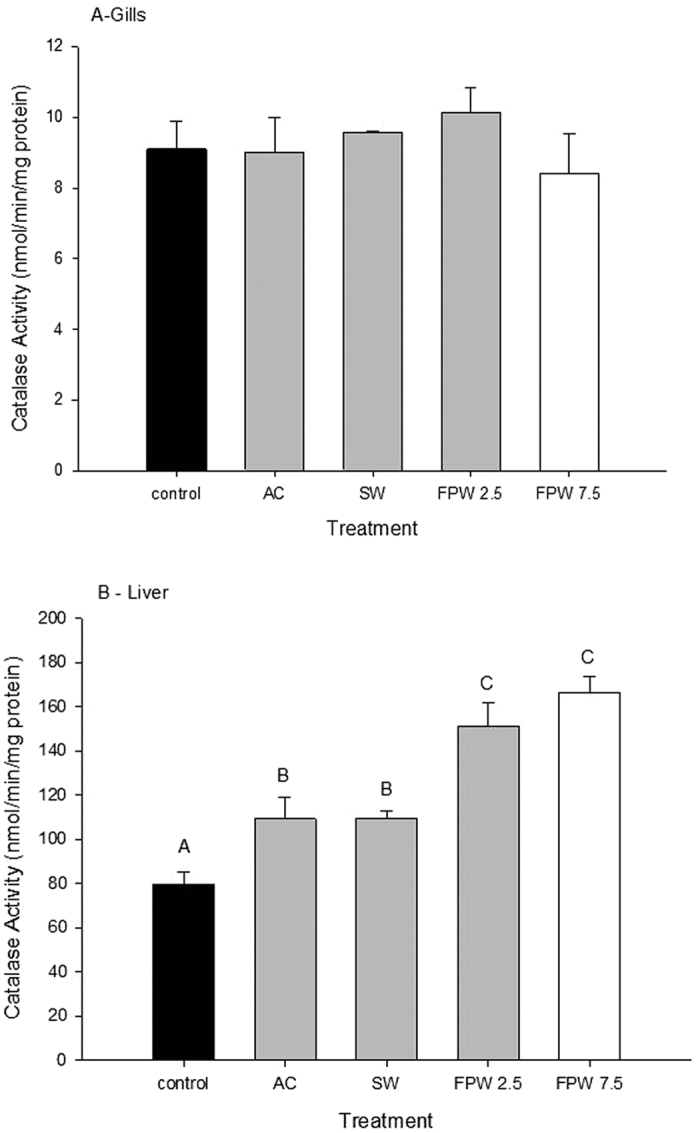
Catalase activity in the gill (**A**) or liver (**B**) of rainbow trout after an acute 48 h exposure to control, activated charcoal treated water (AC), a salt matched control (SW), and flow-back and produced water (FPW 2.5% and 7.5%). Bars represent means (±S.E.M) of 6 to 9 replicates. Bars sharing letters are not significantly (α = 0.05).

**Figure 6:**
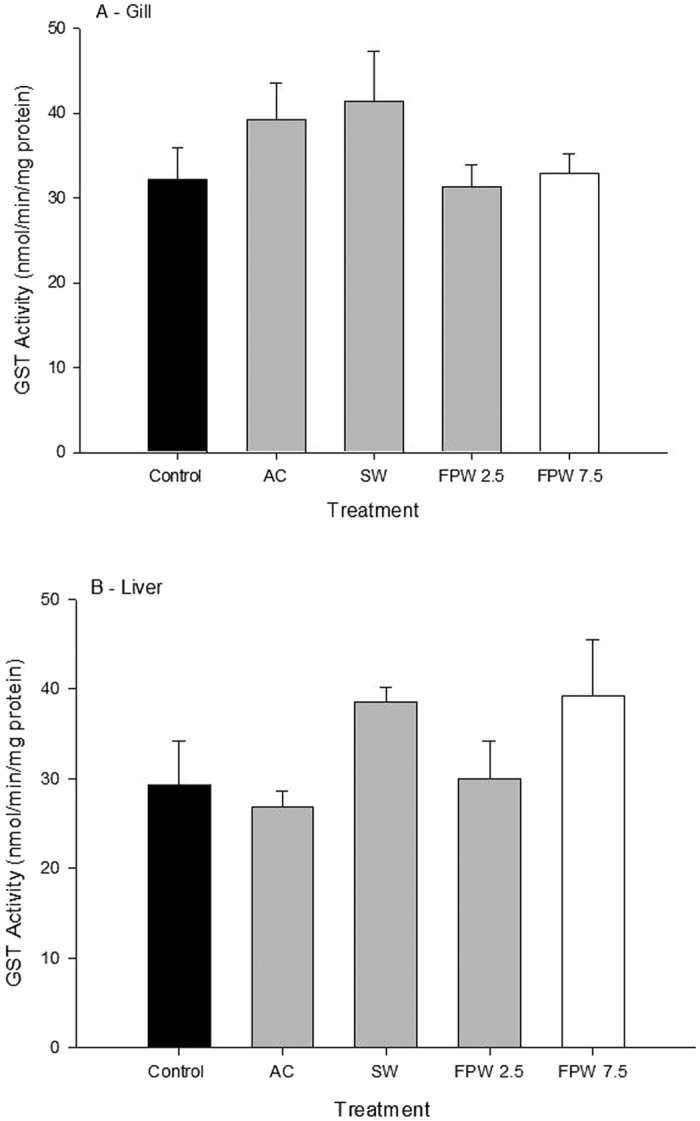
Glutathione —S-transferase activity in the gill (**A**) or liver (**B**) of rainbow trout after an acute 48 h exposure to control, activated charcoal treated water (AC), a salt matched control(SW), and flow-back and produced water (FPW 2.5% and 7.5%). Bars represent means (±S.E.M) of 6 to 9 replicates.

There were no significant changes in catalase activity in gill tissues in any treatment relative to control tissues (Fig. 5A). In contrast, catalase activity in liver showed significant increases in all exposure treatments (p < 0.05; Fig. 5B). Catalase activities in both SW and AC treatments were not significantly different from each other but were significantly elevated from control tissues, with values of 109.4 and 109.5 nmol/min/mg protein, respectively (p < 0.05; Fig. 5B). However, both FPW treatments (2.5% and 7.5%) displayed hepatic catalase activity that was significantly higher than both the SW and AC groups. The highest catalase activity occurred in the 7.5% FPW treatment group at 166.1 nmol/min/mg protein (Fig. 5B). The effect of FPW on liver GST activity is shown in Fig. 6A and B. In both gill and liver there were no significant effects observed.

**Figure 7:**
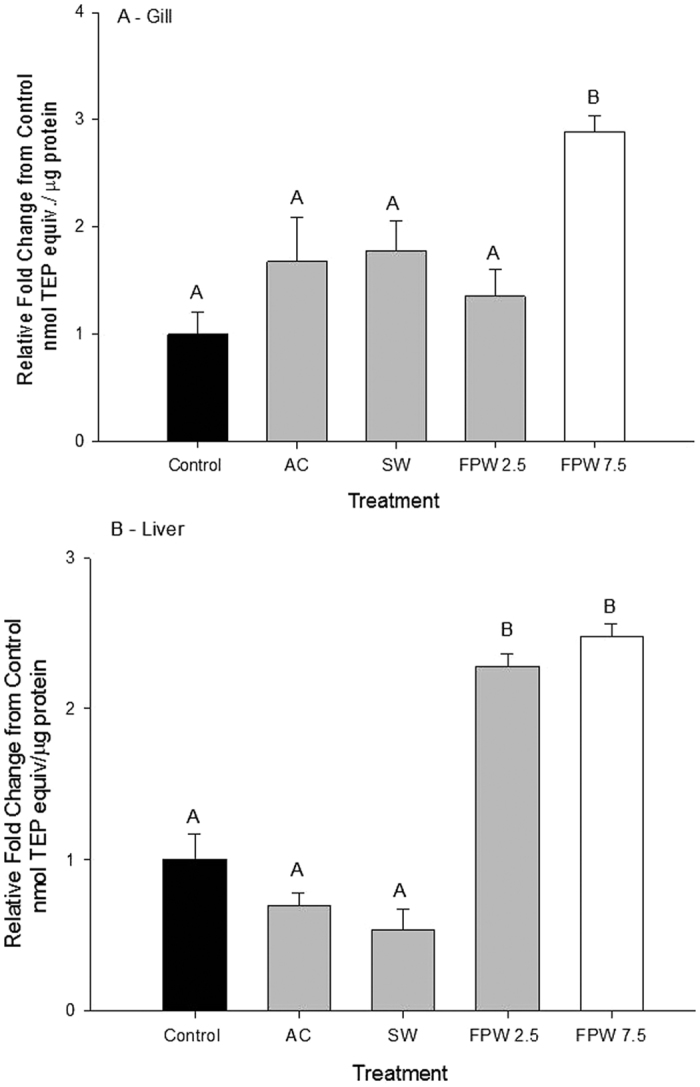
TBARS induction in the gill (**A**) or liver (**B**) of rainbow trout after an acute 48 h exposure to control, activated charcoal treated water (AC), a salt matched control (SW), and flow-back and produced water (FPW 2.5% and 7.5%). Bars represent means (±S.E.M) of 6 to 9 replicates. Bars sharing letters are not significantly (α = 0.05).

There was a significant increase in lipid peroxidation (TBARS) in gill of rainbow trout exposed to 7.5% FPW (p < 0.05). No significant differences were recorded in any other treatments (Fig. 7A). In the liver, 2.5% and 7.5% FPW exposures caused a 2-fold increase in lipid peroxidation relative to control treatments (p < 0.05; Fig. 7B). Neither AC nor SW exposures induced lipid peroxidation.

## Discussion

Acute exposure to FPW generated oxidative stress in the gills and liver, and morphological changes in the gills, of rainbow trout. Changes in gill histology were conserved between AC, SW and FPW treatments, indicating an effect most likely related to high salt exposure. There were interesting differences between gill and liver in terms of oxidative stress, with the liver being particularly sensitive to FPW effects. Activity of cytochrome P450’s (CYPs) was also induced in this tissue, suggesting bioaccumulation and biotransformation occurred and may have played a role in generating oxidative stress in this tissue.

Organic chemical analysis was not performed as part of the current study, although the FPW used was the same as that previously characterized^3^,^4^. These previous studies have reported that this effluent is high in polyaromatic hydrocarbons (PAHs; fluorene and 3,6-dimethylphenanthrene), indicative of a complex mixture. Analysis of the inorganic composition of this FPW^2^ showed that it was high in salts (sodium, calcium, magnesium, potassium, and chloride), and metals (zinc). Previous chemical characterizations of FPW show that the fluids described here are typical of hydraulic fracturing fluids^1^.

Although metals were elevated in full-strength FPW, we believe that they are unlikely to have played a significant role in the observed toxicity. First, the dilution applied (2.5 and 7.5%) would have effectively reduced the concentrations of most metals to levels below detection for our instrumentation (Inductively coupled plasma mass spectrometry). For example, the concentrations of zinc in our study would have ranged from 0.03 (2.5% FPW) to 0.09 μg/L (7.5% FPW), based on the dilution of zinc measured by Blewett and colleagues^2^^2^. Importantly, these concentrations are unlikely to cause harm. These values are well below the 96 h LC_50_ for zinc to rainbow trout (*Oncorhynchus mykiss*) in moderately hard water (66 μg/L^25^). Support for our argument that metals were unlikely to be playing a role in oxidative stress arising from FPW exposure is provided by the lack of differences in any endpoint when comparing the AC and SW treatments. The AC group has similar composition in metals and salts to FPW, while the SW group was made to mimic the salt exposures only.

Gill histology indicated morphological differences in all treatments relative to the control, but no differences between these treatments. Given that each treatment contained elevated concentrations of ions^2^, it is likely that hyperosmotic stress is responsible for the acute morphological changes observed, and that the addition of metals (FPW and AC) and organics (FPW) played little role in alteration of gill morphology. The decreases in ILCM and lamellar width are likely due to osmotic water loss^26^. However, further studies are required to determine whether changes in cell number and/or cell types are also occurring during FPW exposure.

Comparable morphological responses to those found in the current study were shown previously in response to hypersalinity, wherein killifish (*Fundulus heteroclitus*) acclimated to hypersaline water (45 ppt) had a significantly smaller ILCM than fish acclimated to freshwater (1 ppt)^26^. Interestingly, when another salmonid, the Arctic grayling (*Thymallus arcticus*), was exposed to moderately saline water (17 ppt), a rapid gill remodelling occurred in which the ILCM increased dramatically, likely as a protective mechanism^18^. Of note, a study examining the impacts of hydraulic fracturing fluid on two fish species (creek chub and green sunfish) also demonstrated significant impacts on gill morphology^27^,^28^. Epithelial hyperplasia, fusing and swelling of lamella in these species, were in direct opposition to the effects we observed. Individual variation in gill remodelling between fish species, in addition to the complex and variable hydraulic fracturing fluid composition, may explain the differences observed. Furthermore, Papoulias and Velasco^28^ collected fish from the field a month after an FPW leak, and thus the effects observed were likely reflective of a chronic exposure to unknown levels of FPW. Thus the nature of the exposure differed significantly from that employed in the current study (acute exposure to naïve animals). In the present study gill morphology was examined following acute exposure; however, future analyses examining ionoregulation, acid-base regulation, oxygen consumption and waste excretion following AC and FPW exposure are necessary to quantify the physiological impacts of exposure and the subsequent changes in gill morphology.

It is important to note that a reduced ILCM and lamellar width may compromise the protective role of the gill epithelium. Reducing diffusive distance may exacerbate the transport of toxicants such as organic contaminants that may diffuse passively into the gill^29^,^30^. Any impacts of this phenomenon may not be manifested over the acute timeframe of the current study, but could exacerbate effects over a chronic period of exposure. However, if the changes at the gill are related to acute osmotic shock then physiological mechanisms should be enacted to correct this, and thus minimise any long-term impact.

Hepatic EROD activity, which is a catalytic measurement of cytochrome P4501A induction, is a widely used biomarker in fish bioassays for the presence of aryl hydrocarbon receptor (AhR) agonists, including dioxins, polychlorinated biphenyls (PCBs) and PAHs^31^,^32^,^33^,^34^. Our results showed that only in 7.5% FPW was there induction of hepatic EROD (7-fold relative to unexposed control), indicating uptake, and transformation, of organic contaminants from the FPW. These data are consistent with previous research that has shown FPW induces CYP activity in zebrafish and rainbow trout. For example, after 48 hour of exposure to FPW, showed a 2.5-fold induction of EROD activity in rainbow trout liver^3^. PAH’s are likely candidates for this induction, as they occurred at the highest concentrations in the FPW^4^, and they are known to induce EROD activity^31^,^32^,^33^. The liver is particularly sensitive to PAH exposure and subsequent EROD induction as it is the site of PAH accumulation and biotransformation, resulting in metabolites that can be excreted into bile as unconjugated polar metabolites^35^.

There were marked effects of FPW exposure on the oxidative stress response of rainbow trout. When present, these effects were found in all treatment groups relative to control, indicative of salinity as the main driver of effect. However, for some endpoints, FPW treatments exacerbated the effect, suggesting the organic components of the FPW also played a role.

Superoxide dismutase is an enzymatic antioxidant that reduces superoxide anion into hydrogen peroxide in both the mitochondria and the cytosol^36^. Our results show that under an acute salinity stress (SW and AC 7.5%), there was a decrease in SOD activity in the gill and liver (Fig. 4A,B). An effect of salinity on oxidative stress markers has support in the literature. In killifish, SOD activity is salinity-dependent, with activity decreasing in killifish gill with increasing salinity^37^,^38^. This has also previously been seen in sturgeon gradually exposed to increases in salinity, where increases in lipid peroxidation and antioxidant enzymes are observed^39^. While the mechanism of this effect remains elusive, it is known that salinity can cause a variety of physiological changes, including alterations in hormone profiles and energy metabolism, which can contribute to altered cellular oxidative balance^12^.

Although salinity seemed to be important with respect to SOD activity, there was an additional decrease in SOD activity observed in the FPW treatments, suggesting that exposure to organic contaminants further impacted the antioxidant response. In the gills of rainbow trout exposed to both the 2.5% FPW and the 7.5% FPW, a significantly lower SOD activity than the unexposed, SW and AC treatments was seen. This could be attributed to the presence of PAH’s in the FPW. The biotransformation of PAH’s occurs via the cytochrome P450 system^40^. This metabolism directly feeds into the formation of compounds entering redox cycles, and several PAH’s (benzo-a-pyrene, flourene, phenanthrene) cause oxidative stress in aquatic organisms^41^. Previous research has shown a variety of different responses of SOD activity after PAH exposure. Indeed, previous research has shown both increases and decreases after 7 days of exposure to 50 μg/L phenanthrene in goldfish liver tissue^42^. This may help explain the differences in the responses between gill and liver tissues in our study (Fig. 4A,B). There are also many different types of SODs which have different metal cofactors. However, since our assay does not discriminate between these types of SOD and the assay was measuring whole activity, it is possible that certain SOD enzymes (i.e. Cu or Zn SOD), may have been differentially affected.

Catalase is an important antioxidant enzyme that converts the hydrogen peroxide produced from superoxide and decomposes it to oxygen and water. Catalase is found in all tissues, but the liver has the highest basal activity^37^,^43^. Catalase activity responses to FPW exposures were both tissue- and concentration-dependent. The gills of rainbow trout exhibited no change, while the liver showed an increase in catalase activity in response to all treatments (SW, AC, 2.5 and 7.5% FPW). Similar to SOD activity, there was a graded response whereby FPW treatments exhibited greater impacts than SW or AC, suggesting that there were independent effects of both the salinity and the organic toxicants present in the FPW.

The main effects of FPW on catalase activity appear to relate to the increased salt in the water, similar to SOD activity (above). Again, there is precedence for this observation. Blewett and Wood^37^, found that salinity had a marked effect on tissue catalase activity in the gills of killifish. Similarly, the shrimp *Litopenaeus vannamei* subjected to an acute salinity change also displayed altered catalase activity^44^.

Glutathione-S-transferase plays an important role in protecting tissues from oxidative stress and is a critical enzyme in Phase II metabolism. This enzyme acts to conjugate Phase I metabolites, thus transforming lipophilic toxicants, such as PAHs, into hydrophilic compounds for excretion^45^. In the current study, GST showed no change in activity irrespective of treatment (Fig. 6A,B), consistent with the literature. For example, although our FPW was high in PAH’s and therefore might be expected to induce GST activity, only small or non-existent changes in GST activity have been noted in exposures of PAH’s to aquatic biota^35^,^42^,^46^,^47^. Furthermore, GST is not known to be salinity-dependent, previous studies showed no change in gill or liver GST activity in killifish with increasing salinity^48^.

The TBARS assay detects malondialdehyde (MDA), a product formed when lipid membranes are exposed to ROS and damaged^49^. Lipids will exhibit peroxidation if the antioxidant mechanisms that seek to scavenge ROS are impaired and/or unable to cope with an increased production of ROS. Our results show increases in TBARS in our FPW exposures only, indicating an effect probably related to the organic contaminant fraction of the FPW. There are a number of candidate chemicals within the FPW that could give rise to increased lipid peroxidation. These include the lipid peroxide products themselves such as alkoxyls and lipid epoxides which have been shown to induce oxidative stress^50^. Furthermore, other chemicals in fracturing fluid (e.g. naphthalene) are known to increase TBARS formation in the livers of goldfish after exposure^51^. Our data are consistent with our previous work on FPW that showed increases in TBARS in both rainbow trout gill and liver tissue exposed to FPW at similar concentrations^3^. The oxidative stress tissue profiles observed here match tissue profiles in fish exposed to other oil-related contaminants (e.g. crude oil, diesel^52^). Importantly, these data also indicate that although salts alter antioxidant enzyme profiles, these effects do not appear to have consequences insofar as oxidative damage. This suggests that changes in catalase and SOD induced by salinity may differ in mechanism from those resulting from exposure to the organic components of FPW.

## Conclusion

In conclusion, FPW is a complex chemical mixture eliciting a variety of toxicological responses. The greatest biological impact of FPW appears to be via the high salt content with an added stress from the organic component of the FPW. These data suggest a specific signature of FPW exposure suggesting that these changes could be used as biomarkers of exposure and effect, to not only understand the relative impacts of fluid spills, but to determine the zone of impact of each spill, and design post-spill environmental effects monitoring and risk assessment and inform development of best management practices.

## Additional Information

**How to cite this article**: Blewett, T. A. *et al*. The effect of hydraulic flowback and produced water on gill morphology,oxidative stress and antioxidant response in the rainbow trout (*Oncorhynchus mykiss*). *Sci. Rep.* **7**, 46582; doi: 10.1038/srep46582 (2017).

**Publisher's note:** Springer Nature remains neutral with regard to jurisdictional claims in published maps and institutional affiliations.
